# *Notes from the Field:* HIV Diagnoses Among Persons Who Inject Drugs — Northeastern Massachusetts, 2015–2018

**DOI:** 10.15585/mmwr.mm6810a6

**Published:** 2019-03-15

**Authors:** Kevin Cranston, Charles Alpren, Betsey John, Erica Dawson, Kathleen Roosevelt, Amanda Burrage, Janice Bryant, William M. Switzer, Courtney Breen, Philip J. Peters, Tracy Stiles, Ashley Murray, H. Dawn Fukuda, William Adih, Linda Goldman, Nivedha Panneer, Barry Callis, Ellsworth M. Campbell, Liisa Randall, Anne Marie France, R. Monina Klevens, Sheryl Lyss, Shauna Onofrey, Christine Agnew-Brune, Michael Goulart, Hongwei Jia, Matthew Tumpney, Paul McClung, Sharoda Dasgupta, Danae Bixler, Kischa Hampton, Amy Board, Jenifer Leaf Jaeger, Kate Buchacz, Alfred DeMaria

**Affiliations:** ^1^Bureau of Infectious Disease and Laboratory Sciences, Massachusetts Department of Public Health; ^2^Infectious Disease Bureau, Boston Public Health Commission, Boston, Massachusetts; ^3^Epidemic Intelligence Service, CDC; ^4^Division of HIV/AIDS Prevention, National Center for HIV/AIDS, Viral Hepatitis, STD, and TB Prevention, CDC; ^5^Division of Global HIV and TB Prevention, Center for Global Health, CDC; ^6^Division of Viral Hepatitis, National Center for HIV/AIDS, Viral Hepatitis, STD, and TB Prevention, CDC; ^7^Oak Ridge Institute for Science and Education, Oak Ridge, Tennessee.

From 2000 to 2014, the number of annual diagnoses of human immunodeficiency virus (HIV) infection in Massachusetts declined 47% (*1*). In August 2016, however, the Massachusetts Department of Public Health (MDPH) received reports of five new HIV cases among persons who inject drugs from a single community health center in the City of Lawrence (*2*). On average, less than one case per month among persons who inject drugs had been reported in Lawrence during 2014–2015 from all providers. Surveillance identified additional cases of HIV infection among such persons linked to Lawrence and Lowell, in northeastern Massachusetts, during 2016–2017. In 2018, MDPH and CDC conducted an investigation to characterize the outbreak and recommend control measures.

Investigators reviewed surveillance data and HIV-1 polymerase (*pol*) gene nucleotide sequences derived from drug resistance testing and interviewed persons with HIV infection in northeastern Massachusetts. Cases were defined as diagnoses of HIV infection in northeastern Massachusetts during January 2015–May 2018 in 1) a person who injects drugs who received medical care, experienced homelessness, resided, or injected drugs in Lawrence or Lowell; 2) a person who was epidemiologically linked as an injecting or sex partner of a person with HIV infection connected to Lawrence or Lowell; or 3) a person with an HIV-1 *pol* nucleotide sequence molecularly linked at a genetic distance of ≤1.5% (as determined by pairwise sequence analysis) to that of another person in the investigation who was connected to Lawrence or Lowell. Qualitative interviews were conducted with a purposeful sample of 34 persons who inject drugs to assess risk factors for HIV infection and with 19 clinicians and other stakeholders in Lawrence and Lowell to identify available medical and social services.

As of June 30, 2018, a total of 129 persons meeting the case definition were identified; 74 (57%) were male, 94 (73%) were aged 20–39 years at diagnosis, 87 (67%) were non-Hispanic white, and 38 (29%) were Hispanic. Most (114; 88%) reported a history of injection drug use (Figure), including four (3%) who also reported male-to-male sexual contact; 116 (90%) had laboratory evidence of past or current hepatitis C virus infection. Median CD4+ cell count at diagnosis was 550 cells/*μ*L (range = 1–1,470), suggestive of a number of recent infections (*3*). Molecular analysis aided case identification: 28 (22%) cases had epidemiologic links only; 69 (53%) had both epidemiologic and molecular links; and 32 (25%) had molecular links only. Four clusters of ≥5 cases were identified using molecular links; two of these clusters accounted for 78 (60%) cases.

**FIGURE:**
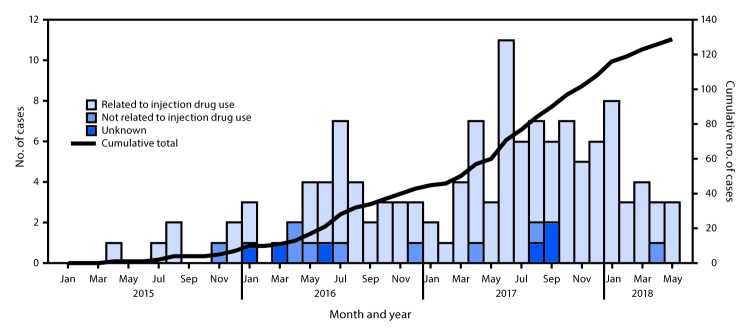
Human immunodeficiency virus diagnoses linked to Lawrence and Lowell, Massachusetts, January 2015–May 2018

In qualitative interviews, the 34 persons who inject drugs variously identified opioids alone, stimulants (i.e., cocaine and methamphetamine) alone, or both opioids and stimulants as their drugs of choice. Sharing syringes and other equipment, experiencing homelessness, being incarcerated, or exchanging sex for drugs during the previous year also were reported. Stakeholders reported that fentanyl had replaced heroin in local communities, was cheaper in Lawrence than in other cities in the region, and had increased injection frequency. The reported increased frequency of fentanyl injection might have increased transmission in Lawrence and Lowell. Stakeholders also reported that frequent homelessness and incarceration among injection drug users undermined HIV treatment success because of interrupted treatment, missed appointments, and having multiple care providers. An additional challenge noted was syringe services program (SSP) accessibility. Lowell had a privately funded SSP with limited days and hours of operation; since 2017, Lawrence had a state-funded SSP with daily availability, but no weekend or evening hours.

Opioid overdose deaths have increased rapidly in Lawrence and Lowell since 2013 (*4*), with postmortem fentanyl detection increasing statewide (*5*). The presence of multiple molecular clusters and unlinked infections suggests multiple introductions of HIV among persons who inject drugs as well as recent and rapid transmission in the context of some longstanding HIV infections.

Lawrence and Lowell approved state-funded SSPs in 2016 and 2018, respectively. MDPH has since deployed additional field staff members to link persons with HIV infection to care and to provide partner services. MDPH and local partners are expanding services that address social instability attributable to homelessness and incarceration and increase knowledge about safer injection practices among persons who inject drugs. MDPH will continue HIV testing, field investigation, and molecular cluster detection and response statewide.
